# Analysis of Breast Cancer Based on the Dysregulated Network

**DOI:** 10.3389/fgene.2022.856075

**Published:** 2022-02-15

**Authors:** Yanhao Huo, Xianbin Li, Peng Xu, Zhenshen Bao, Wenbin Liu

**Affiliations:** ^1^ Institute of Computational Science and Technology, Guangzhou University, Guangzhou, China; ^2^ School of Computer Science of Information Technology, Qiannan Normal University for Nationalities, Duyun, China

**Keywords:** breast cancer, dysregulated network, cancer-related pathways, driver genes, drug targets, survival analysis

## Abstract

Breast cancer is a heterogeneous disease, and its development is closely associated with the underlying molecular regulatory network. In this paper, we propose a new way to measure the regulation strength between genes based on their expression values, and construct the dysregulated networks (DNs) for the four subtypes of breast cancer. Our results show that the key dysregulated networks (KDNs) are significantly enriched in critical breast cancer-related pathways and driver genes; closely related to drug targets; and have significant differences in survival analysis. Moreover, the key dysregulated genes could serve as potential driver genes, drug targets, and prognostic markers for each breast cancer subtype. Therefore, the KDN is expected to be an effective and novel way to understand the mechanisms of breast cancer.

## Introduction

According to global cancer statistics in 2020, Breast cancer has become the most common cancer, with 2.3 million new cases (Sung et al., 2021). As a heterogenetic malignancy, breast cancer can be classified into four subtypes: Luminal A, Luminal B, Basal-like, and Her2-enriched (Cheang et al., 2009; Inic et al., 2014). Although significant improvements have been achieved, a better understanding of genetic changes will lead to better diagnosis and treatment of this disease (Liang et al., 2021).

The genetic variation of driver genes has been considered as one of cancer’s most critical intrinsic factors (Akhavan-Safar et al., 2021). Thus, many computational tools have been developed to identify potential driver genes. For example, MaxDriver developed by Chen et al. detect driver genes based on the maximum information flow in the heterogeneous network (Chen et al., 2013). DawnRank can directly prioritize the driver genes at the individual patient level (Hou and Jian, 2014). And Shi et al. proposed a network diffusion method to identify driver genes (Shi et al., 2016). Among these tools, DriverNet is probably the most competitive tool which considers both gene mutation and abnormal expressions of downstream genes (Bashashati et al., 2012).

Differentially expressed genes (DEGs) analysis is used to identify potential biomarkers or prognostic markers for breast cancer (Yang et al., 2019). Based on DEGs and the survival analysis of hub genes in protein-protein interaction network (PPI), Wu et al. identified that ESR1 and PGR may be potential prognostic markers of ER-positive breast cancer (Wu et al., 2020). Huan et al. found that estradiol (E2) is a biomarker of breast cancer based on the analysis of DEGs in the PPI network (Huan et al., 2014). Furthermore, Eskandari et al. constructed a gene regulatory network by common DEGs to identify the key therapeutic targets for each subtype of breast cancer (Eskandari and Motalebzadeh, 2019).

In summary, previous works have started from the single and independent abnormal expression of genes, but ignored the importance of changes in the interactions between genes. Actually, cancer occurs because of abnormal interactions between genes that lead to their abnormal expressions (Peng et al., 2012; Gao et al., 2013; Bao et al., 2016; Bao et al., 2020; Chai et al., 2022). In this paper, we propose a new way to measure the regulation strength between genes based on their relative expression values. Then the dysregulated network (DN) can be determined by the dysregulated interactions between normal and disease samples. Results show that not only is the key dysregulated network (KDN) enriched in many potential breast cancer related-pathways and important driver genes, but is also closely related to drug targets. Therefore, the proposed KDN provides a new tool for elucidating the underlying mechanism and potential drug repurposing for breast cancer.

## Materials and Methods

### Materials

Both the gene expression dataset and genomic aberration dataset are downloaded from https://xenabrowser.net/datapages/. Gene expression dataset includes Luminal A, Luminal B, Basal-like, and HER2-enriched subtypes. Genomic aberration dataset includes gene-level copy number alteration and somatic mutation (SNP and INDEL). The somatic mutation dataset is a binary matrix containing the gene-level non-silent mutation. The influence network includes directed gene interactions from KEGG, Reactome, Panther, CellMap, and NCI Pathway Interaction Databases (Wu et al., 2010). The 29 targeted drugs are downloaded from https://www.cancer.gov/about-cancer/treatment/drugs/breast (National Cancer Institute), and their corresponding targets are obtained from https://clue.io/repurposing-app. Table 1 presents the details of the datasets and network including the number of genes, the number of samples, and the number of interactions.

**TABLE 1:** The details of datasets and network.

Datasets			Number of genes	Number of samples	Number of interactions
Gene expression (TCGA-BRCA)	Normal	—	34,127	99	—
	Tumor	Luminal A		225	—
		Luminal B		123	—
		Basal-like		97	—
		HER2-enriched		57	—
Genomic aberrations	—	gene-level copy number alteration	24,776	1081	—
		somatic mutation (SNP and INDEL)	40,543	792	—
Influence network	—	—	9728	—	146171

## Methods

From the perspective of gene regulatory network, it is the significantly abnormal interaction between genes that pushes cells operating from normal state to disease state. Therefore, analysis of dysregulation may help to reveal more biological insights than traditional differentially expressed genes (DEGs). Our motivation is that an upstream gene will have more influence on its downstream genes if the expression of the former is larger than that of the latter, and vice versa. Therefore, we define the regulation strength of gene i to gene j as

$$r_{ij}=\log\frac{g_{i}}{g_{j}}$$ (1)

where $g_{i}$ and $g_{j}$ is the expression value of gene i and gene j respectively. In this paper, we name the network composed of the dysregulated interactions as the dysregulated network (DN).

Then, the average absolute difference of the dysregulated strength $ds_{ij}$ of gene i to gene j can be calculated as

$$ds_{ij}=\left|{\bar{r}}_{ij}^{D}-{\bar{r}}_{ij}^{N}\right|$$ (2)

where ${\bar{r}}_{ij}^{D}$ and ${\bar{r}}_{ij}^{N}$ denote the average regulation strength in the disease and normal state, respectively. Further, the dysregulation score $d_{i}$ of gene i is defined as the sum of the dysregulated strength to all its downstream genes

$$d_{i}=\sum_{j=1}^{n_{i}}ds_{ij}$$ (3)

where $n_{i}$ is the number of the direct downstream genes of gene i . A higher $d_{i}$ indicates that gene i regulate more downstream genes with the higher $ds_{ij}$ , otherwise, gene i regulate less downstream genes with the lower $ds_{ij}$ . Finally, based on the key genes and their dysregulated interactions, the key dysregulated network (KDN) is obtained.

**FIGURE 1:**
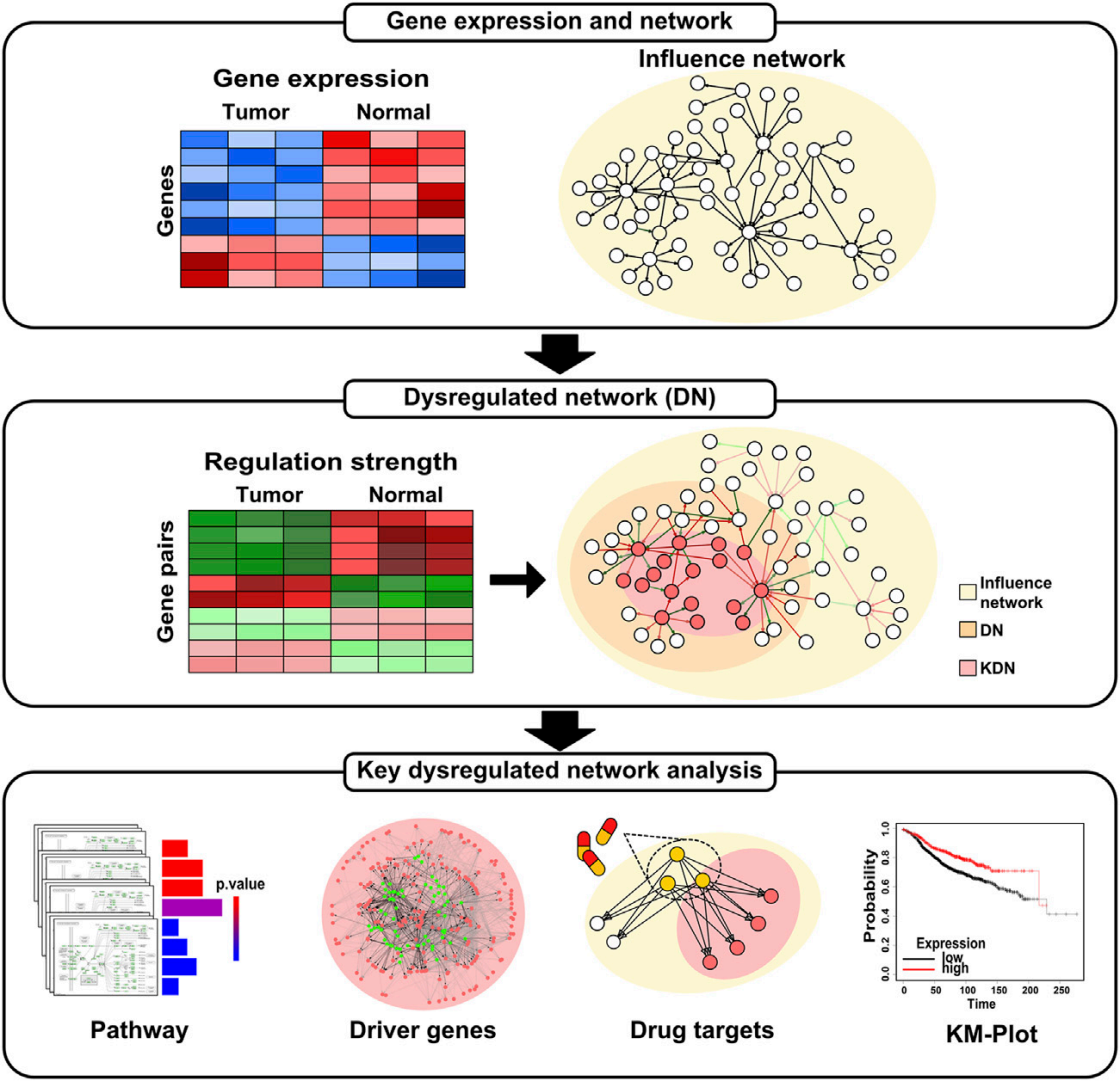
Overview of the analysis workflow.

Figure 1 shows an overview of the analysis workflow for this study. First, we construct the DN based on gene expression data and influence network. Then, we identify the KDN of each subtype. Finally, we conduct pathway enrichment analysis, driver genes analysis, drug targets enrichment analysis, and survival analysis for the obtained KDN.

### Identifying Driver Genes through DriverNet

Driver genes can be manifested through the outlying expression of genes in influence network. So, Bashashati et al. (Bashashati et al., 2012) developed a computational framework called DriverNet to identify the potential driver genes effectively. In DriverNet, a bipartite graph is constructed through genomic aberrations matrix, outlier matrix, and influence network. And, based on the bipartite graph, DriverNet could rank the genes according to the number of events (outliers). Then, a set of potential driver genes is obtained.

## Results and Discussion

### The Dysregulated Network

For each subtype, the dysregulated interactions are determined with p-value≤10E−4 and ds≥2 by Limma package in R. Table 2 presents the four dysregulated networks (DNs), including the number of genes, the number of interactions, the average degree, and the average betweenness of genes. Only about 50% of the genes and 20% of the interactions from the background influence network constitute the DN. The average degree ( ≈8 ) and betweenness ( ≈8000 ) indicate the DN is highly interconnected.

**TABLE 2:** Overview of the DNs.

Subtypes	Number of genes	Number of interactions	Average degree	Average betweenness
Luminal A	4971	18,771	7.28	6875
Luminal B	5847	26,853	9.29	8374
Basal-like	5573	23,307	8.36	8304
HER2-enriched	5679	24,483	8.62	8412

**FIGURE 2:**
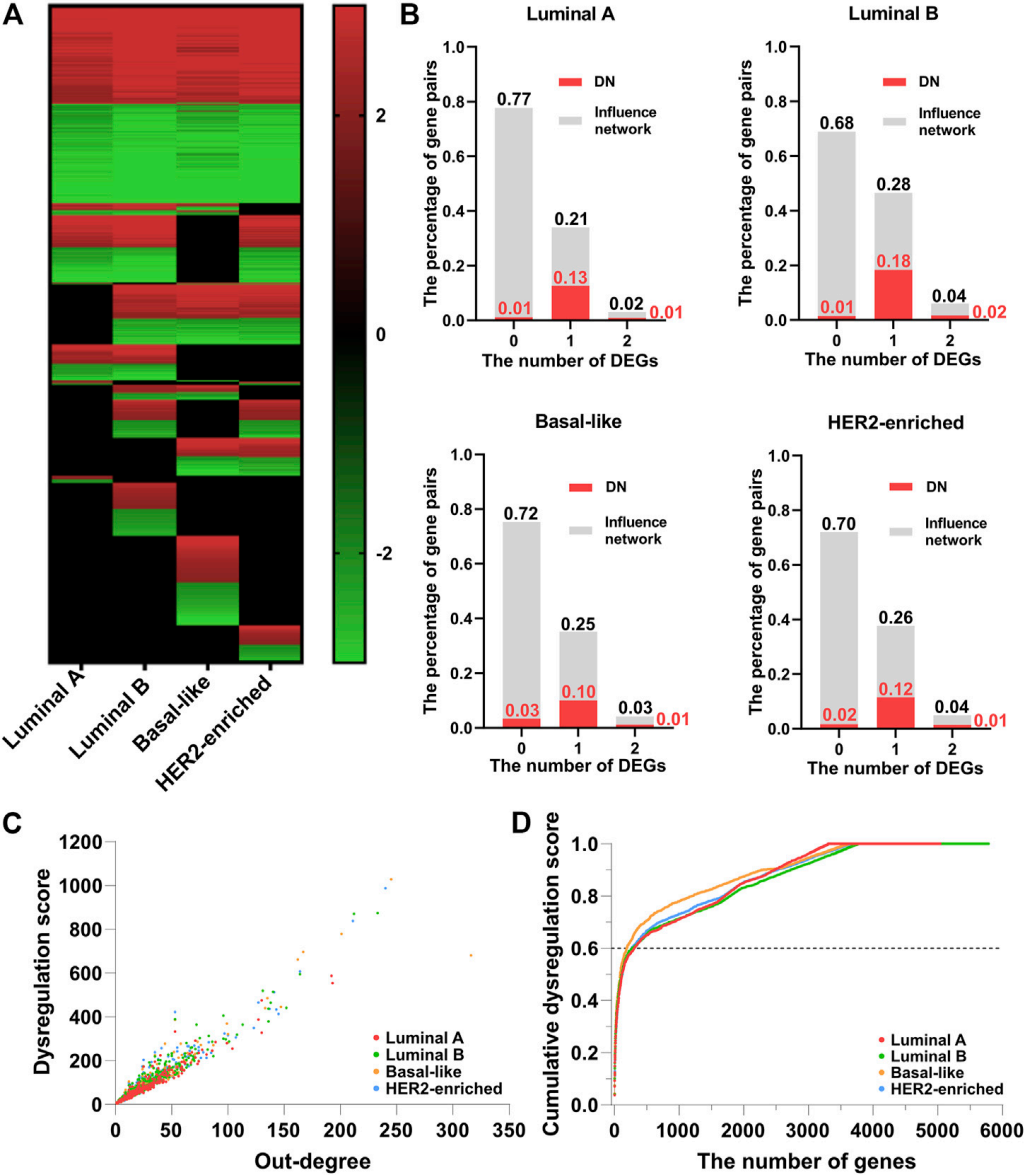
The dysregulated network (DN) of breast cancer. **(A)** The heatmap of the dysregulated interactions in the four breast cancer subtypes. **(B)** The percentage of interactions with 0, 1, and 2 DEGs (gray color) and dysregulated interactions (red color) in the background influence network. **(C)** The scatter plot of the dysregulation score and out-degree of genes in the DN. **(D)** The relationship between the cumulative dysregulation score and the number of genes in the DN.

Figure 2A shows the heatmap of the dysregulated interactions in the four breast cancer subtypes. The interactions are ordered according to their observed frequency in subtypes. We color the interaction red when the disease state has a higher average regulation strength of interactions, and green otherwise. Black at the bottom represents that the interactions are not significantly dysregulated in the corresponding subtype. About 50% of the dysregulated interactions are shared by the four subtypes, and the overlapped interactions have the same dysregulation pattern. The abnormal regulation in these gene pairs may form the common mechanisms of the four subtypes. On the other hand, about 10% of the dysregulated interactions appear in only one subtype which may characterize the different phenotypes of the four subtypes at network level. Therefore, the corresponding DN may contribute to the development of the four subtypes of breast cancer.

As the regulation strength measures the dysregulated interactions, it is natural to ask if the dysregulated interactions are essentially caused by differentially expressed genes (DEGs). To examine the impact of DEGs ( p-value≤10E−4 and FC≥1.5 ) on the dysregulated interactions ( p-value≤10E−4 and ds≥2 ), Figure 2B shows the percentage of interactions with 0, 1, and 2 DEGs in the background network (gray color). And the percentage of dysregulated interactions in each group is shown with red color. Obviously, most of the dysregulated interactions, 40%–65%, come from the group with just one DEG; only a few come from the other groups. In sum, the DN only contains about half of the DEGs. Given that gene i regulates gene j , their interactions may not be significantly abnormal if their expressions change in the same way, even if one or two of them are DEGs. On the other hand, the interactions may be significantly abnormal if their expressions change inversely, even if both are not significantly differentially expressed. Therefore, dysregulated interactions can reveal the regulation abnormality of subtypes, which is hard for DEGs to detect.

Figure 2C shows the scatter plot of the dysregulation score $d_{i}$ of gene i and its outdegree in the DN. First, the dysregulation score is linearly proportional to the outdegree: genes with larger outdegree tend to have larger dysregulation scores. These genes may play important roles in the DN. Secondly, only a few genes have extraordinarily large dysregulation scores. In order to determine the key genes in the DN, we sort them in descending order according to their dysregulation score. Then, the dysregulation score is normalized by the total score of all genes and Figure 2D shows the curve of the accumulative normalized dysregulation score, which is sorted by gene order. This curve grows rapidly at first and then increases slowly as the cumulative score reaches 60%. The genes contributing to the 60% cumulative dysregulation score only include about 5% of genes in the DN (306, 274, 189, and 267 genes for Luminal A, Luminal B, Basal-like, and HER2-enriched subtypes respectively). In this paper, we refer to the network of these key genes and their dysregulated interactions as the key dysregulated network (KDN).

### Key Dysregulated Genes are Enriched in Critical Breast Cancer-Related Pathways

To investigate the biological functions of the KDN, we conduct a pathway enrichment analysis on key dysregulated genes with p-value≤0.05 . We also take the same analysis of the top 300 differentially expressed genes (DEGs) obtained by Limma package. Table 3 lists the top 30 enriched pathways by key genes and DEGs. Key genes are significantly enriched in many well-known breast cancer-related pathways including Pathway in cancer, Ras signaling pathways, MAPK signaling, Estrogen signaling, Breast cancer, Prolactin signaling pathways, etc. However, the top 300 ordinary DEGs are only enriched in very few pathways which are not the critical ones in breast cancer. This comparison suggests that the genes in the KDN are more biologically related to breast cancer than DEGs.

**TABLE 3:** Top 30 enrichment pathways of key genes and enrichment pathways of DEGs.

Pathway	*p*-value			
Top 30 enrichment pathways of key genes	Luminal A	Luminal B	Basal-like	HER2-enriched
Pathways in cancer	5.55E-40	4.90E-43	1.31E-35	4.20E-45
PI3K-Akt signaling pathway	1.05E-38	4.04E-34	8.13E-24	9.65E-41
Relaxin signaling pathway	1.33E-33	2.68E-31	7.43E-17	6.93E-31
Ras signaling pathway	4.66E-25	1.69E-32	6.26E-16	1.11E-27
Chemokine signaling pathway	4.28E-27	1.81E-19	2.28E-15	4.21E-21
Dopaminergic synapse	8.82E-26	3.41E-21	2.33E-14	7.04E-21
Focal adhesion	3.62E-20	1.07E-19	7.65E-14	2.66E-21
MAPK signaling pathway	9.99E-23	6.18E-26	9.58E-14	1.71E-24
Human cytomegalovirus infection	5.45E-20	1.14E-20	1.77E-13	3.04E-21
Human papillomavirus infection	5.06E-21	2.51E-17	4.80E-13	1.49E-18
Cholinergic synapse	3.94E-17	1.51E-13	1.32E-12	1.88E-16
Kaposi sarcoma-associated herpesvirus infection	2.30E-15	2.17E-19	2.12E-12	2.12E-14
Hepatitis B	5.04E-22	3.71E-20	2.79E-12	7.95E-20
cAMP signaling pathway	5.31E-18	4.87E-14	4.07E-12	3.65E-18
Circadian entrainment	7.08E-16	4.90E-12	3.27E-15	6.26E-14
Human T-cell leukemia virus 1 infection	3.46E-15	7.47E-14	5.72E-12	3.89E-16
Proteoglycans in cancer	9.06E-28	2.45E-22	9.00E-12	3.86E-17
Estrogen signaling pathway	1.45E-13	2.33E-12	7.87E-12	5.46E-16
Lipid and atherosclerosis	1.82E-15	5.79E-15	2.82E-11	1.17E-14
Thyroid hormone signaling pathway	2.68E-12	8.07E-15	5.89E-11	1.35E-12
IL-17 signaling pathway	1.59E-18	1.76E-14	6.83E-11	2.90E-14
Amphetamine addiction	9.51E-18	6.48E-12	6.34E-11	3.03E-15
Parathyroid hormone synthesis, secretion and action	5.32E-18	3.33E-11	5.24E-11	1.51E-19
AGE-RAGE signaling pathway in diabetic complications	8.22E-19	1.86E-19	2.35E-09	5.15E-18
Breast cancer	1.56E-16	1.50E-19	2.40E-09	3.57E-15
Human immunodeficiency virus 1 infection	4.08E-10	3.67E-15	7.31E-09	3.66E-13
Gastric cancer	1.55E-13	2.84E-17	2.44E-08	4.72E-14
Melanogenesis	1.48E-17	1.12E-10	2.77E-08	1.81E-12
Cocaine addiction	1.30E-12	5.58E-10	2.90E-08	7.37E-15
Rap1 signaling pathway	2.55E-20	6.69E-22	3.80E-08	1.64E-21
Growth hormone synthesis, secretion and action	5.34E-23	3.08E-18	3.81E-08	1.67E-22
Oocyte meiosis	7.24E-10	8.85E-08	1.64E-13	4.16E-10
Melanoma	1.99E-09	7.96E-14	2.66E-07	2.25E-11
Oxytocin signaling pathway	3.98E-13	3.26E-07	6.92E-10	5.36E-07
Osteoclast differentiation	1.09E-11	2.93E-17	8.64E-07	4.48E-11
Prolactin signaling pathway	3.78E-15	2.65E-15	1.95E-06	6.51E-14
Longevity regulating pathway	1.78E-20	8.69E-11	3.44E-06	1.13E-12
Morphine addiction	1.47E-08	4.55E-06	4.84E-10	1.32E-07
TNF signaling pathway	7.43E-23	9.76E-16	7.15E-06	1.93E-14
ErbB signaling pathway	2.21E-16	1.93E-14	1.62E-05	4.70E-12
Prion disease	1.29E-04	1.02E-11	4.29E-06	1.70E-14
Insulin resistance	7.64E-19	5.24E-13	1.16E-03	5.36E-09
Proteasome	—	5.97E-13	1.41E-12	6.89E-17
Parkinson disease	—	1.95E-05	8.59E-10	1.15E-09
Enrichment pathways of DEGs				
Cell cycle	2.32E-02	2.25E-14	1.44E-24	6.49E-13
Progesterone-mediated oocyte maturation	5.08E-03	1.20E-04	1.76E-07	1.47E-04
Oocyte meiosis	—	2.38E-07	3.89E-09	3.19E-06
Cellular senescence	—	3.39E-02	3.94E-05	—
Human T-cell leukemia virus 1 infection	—	5.87E-05	—	—
Homologous recombination	—	—	3.03E-03	—

**FIGURE 3:**
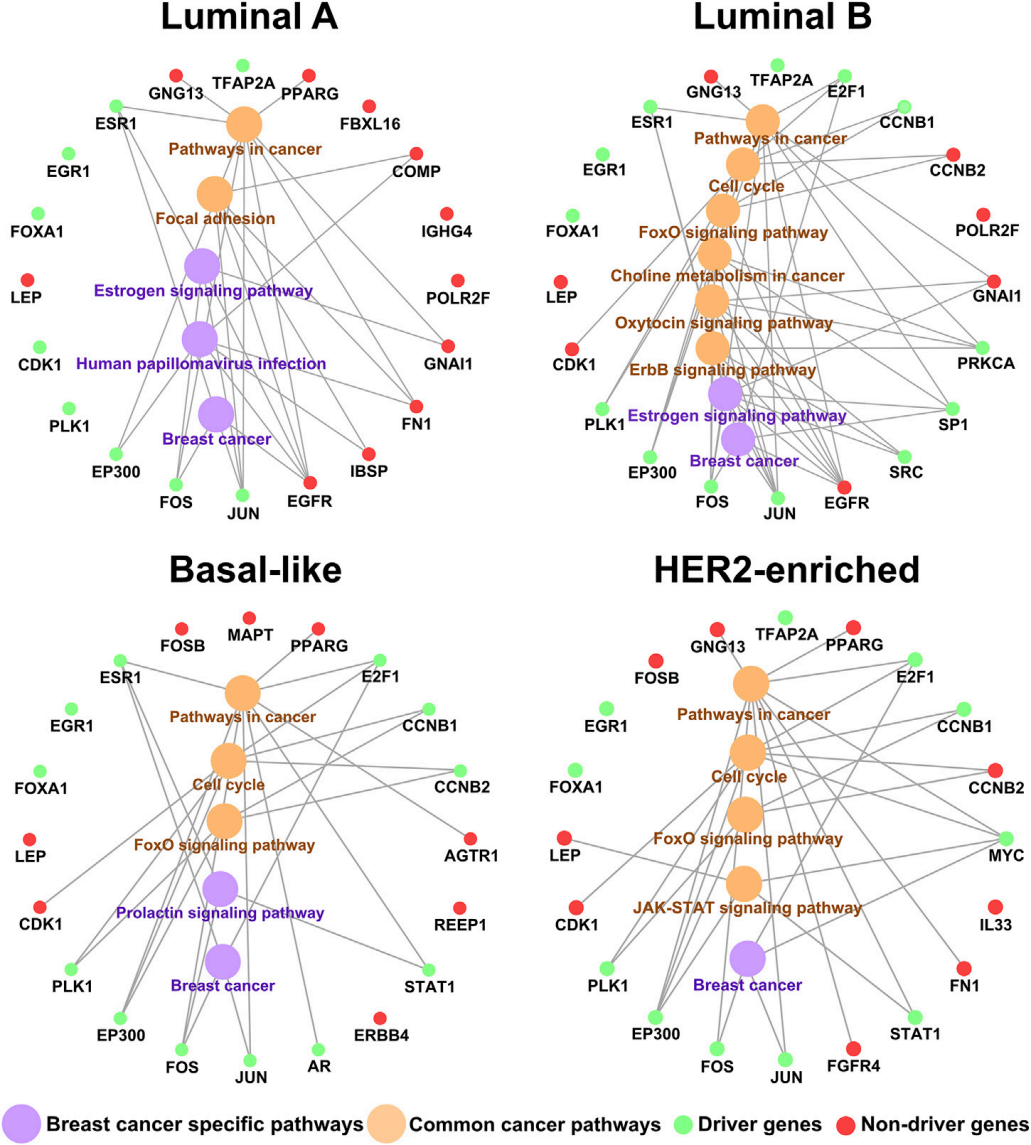
The relation of the top 20 key genes and their enriched pathways.

Furthermore, we take an enrichment analysis of the top 20 genes in the KDN. Figure 3 shows the relation of the top 20 key genes and their enriched pathways by p-value≤0.05 . Green and red colors denote driver genes and non-driver genes respectively. Yellow and purple colors denote common cancer pathways and breast cancer specific pathways. Surprisingly, even the top 20 key genes are significantly enriched in some breast cancer specific pathways. Among them, the breast cancer pathway contains four subpathways and connects with many important signaling pathways, such as MAPK pathway, PI3K-Akt pathway, Notch signaling pathway, Wnt signaling pathway, P53 signaling pathway, Cell cycle pathway, etc. For the Estrogen signaling pathway, Tang et al. reported that Estrogen-triggered signaling cascades play an important role in the initiation and development of most human breast cancer (Song and Santen, 2006). In addition, Kitajima et al. also reported that Estrogen and its receptor can regulate the development and progression of breast cancer in most cases (Shin-Ichi et al., 2010). For the Prolactin signaling pathway, a 20-years prospective study has shown that Prolactin can promote proliferation and cell motility in later stage breast tumor development (Tworoger and Hankinson, 2006; Tworoger et al., 2013). For the Human papillomavirus infection pathway, HR-HPV DNA infection exists in breast cancer tissue, thus closely related to the occurrence and development of breast cancer (Wang et al., 2009). And, these pathways have crosstalk with other common cancer pathways (Chen and Wang, 2012; Sato, 2013). For example, Pathways in cancer, Focal adhesion (Ocak et al., 2010; Gari et al., 2016; Gu et al., 2018; Gong et al., 2019), Cell cycle, FoxO signaling pathway (Mohd et al., 2017; Gong et al., 2020), Choline metabolism in cancer, Oxytocin signaling pathway (Cassoni et al., 1994; Pequeux, 2002; Wang et al., 2020), ErbB signaling pathway (Liu et al., 2008; Aline et al., 2015), and JAK-STAT signaling pathway (Hernández-Vargas et al., 2011; Wang et al., 2018; Na and Balko, 2019).

Based on the biological functions, the driver genes (Bashashati et al., 2012) in common top 20 key genes are highly associated with breast cancer subtypes. As shown in Figure 3, EGR1, EP300, FOS, JUN, FOXA1, PLK1, ESR1, and E2F1 are the driver genes for corresponding subtype. As a tumor-suppressor gene in breast cancer, overexpression of EGR1 in breast tumor cells markedly reduces transformed growth and tumorigenicity (Huang et al., 1997; Ronski et al., 2010). EP300 is recruited by the estrogen receptor alpha, a hormone inducible transcription factor, to mediate the mitogen effect of the ovarian steroid estrogen, which is a strong risk factor for breast cancer development (Wirtenberger et al., 2006). The FOS family is one of the AP-1 transcription factors, which regulated many proteins involved in breast cancer invasion (Milde-Langosch et al., 2004). Activated JUN is predominantly expressed at the invasive front in breast cancer and is associated with proliferation and angiogenesis (Vleugel et al., 2006). FOXA1 can influence the expression of a large number of genes in breast cancer associated with metabolic processes, regulation of signaling, and the cell cycle (Wolf et al., 2005). PLK1 mediates estrogen receptor (ER)-regulated gene transcription in human breast cancer cells. And PLK1-coactivated genes include classical ER target genes such as Ps2, Wisp2, and Serpina3 and are enriched in developmental and tumor-suppressive functions (Wierer et al., 2013). ESR1 encodes estrogen receptor-α, which is a major biomarker in the development of breast cancer (Yang et al., 2021). E2F1 expression is regulated by the estrogen receptor *α* (ERα) to mediate tamoxifen resistance in ERα-positive breast cancer cells (Montenegro and Cancer, 2014). And E2F1 can drive the metastasis of breast cancer (Hollern et al., 2019).

### Driver Genes are Enriched in the Key Dysregulated Network

At the genomic level, driver genes are considered to be one of the most important factors in cancer initiation and progression. The driven mutations in the genome provoke abnormal function at protein level and impact the expression of the downstream genes. Therefore, driver genes, as an intrinsic driven regulation mechanism, should also play a critical role in the obtained DN. We apply DriverNet to identify driver genes, and it identifies 205, 154, 249, and 147 driver genes for Luminal A, Luminal B, Basal-like, and HER2-enriched subtypes respectively. We find that about 90% of the determined driver genes are observed in the dysregulation network.

**FIGURE 4:**
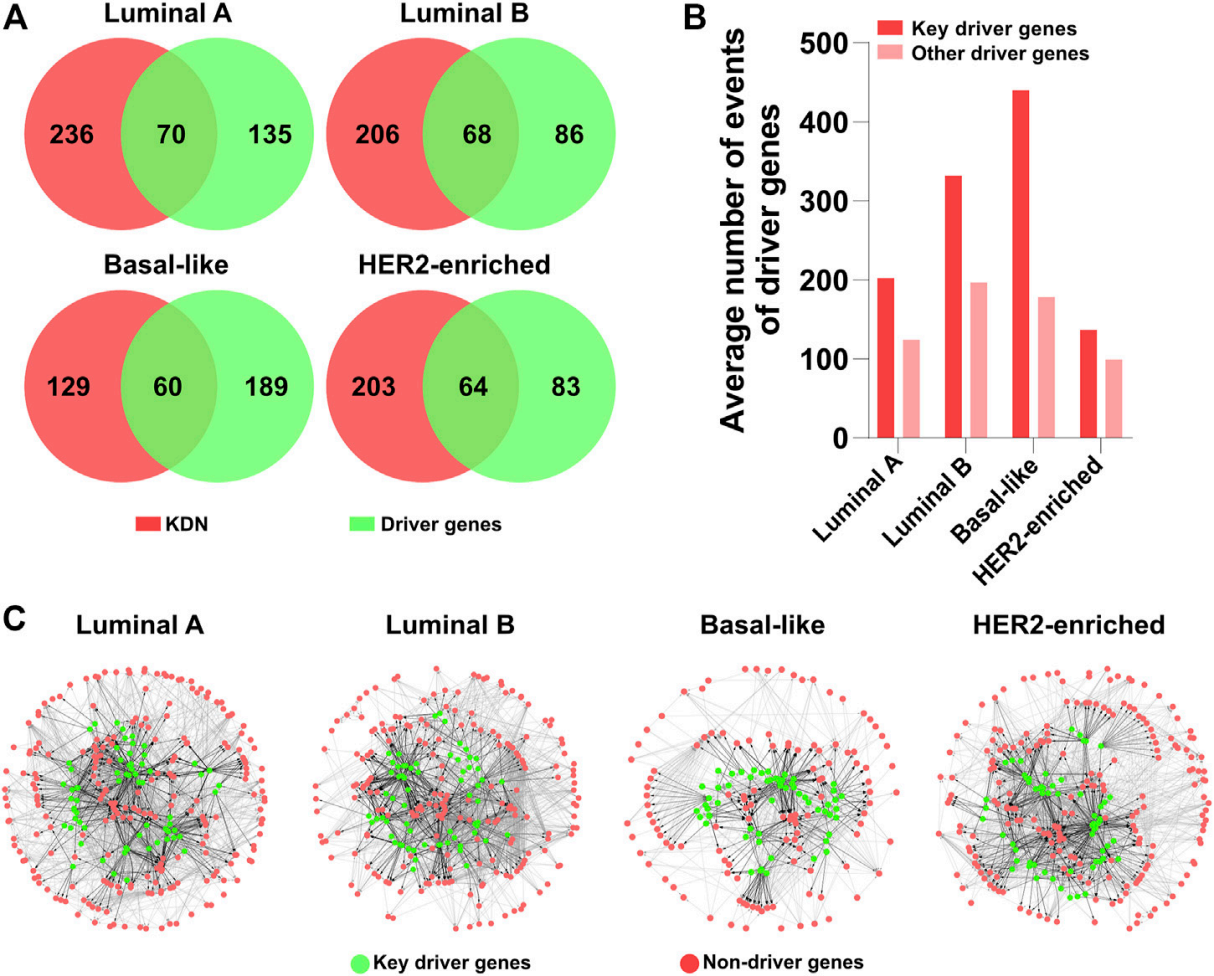
Driver gene analysis. **(A)** Venn diagrams of the key genes and driver genes. **(B)** The average number of events of key driver genes and other driver genes. **(C)** KDN with driver genes in green color.

As the key genes in DN constitute most of the dysregulation consequences, we are interested in the driver genes in the KDN. Figure 4A shows the Venn diagrams of the key genes and the identified driver genes in the four subtypes. In the KDN, about 20–30% of the genes are driver genes. That is, the KDN is enriched with a larger portion of driver genes. Furthermore, Figure 4B shows the driver genes’ average number of events as defined by DriverNet (Bashashati et al., 2012), whether they are in the KDN or not. The former’s average number of events is obviously higher than that of the latter, which demonstrates that key driver genes explain more abnormal expressed genes in the patient group than the latter.

In Figure 4C, green and red denote whether they are driver genes; black and gray denote whether the interactions originate from driver genes. The KDNs are highly connected in the central part which include some driver genes and their downstream genes, while the peripheral part is relatively sparsely connected which includes only non-driver genes. Therefore, we may hypothesize that these driver genes in the central part constitute the core tumorigenesis genes. Their mutations are the major causal factors to the corresponding subtypes. These driver genes first exert their abnormal effects on their direct downstream genes. And the downstream genes propagate the abnormal signals to other peripheral genes. Finally, the interactions between genes in the KDN contribute to the initiation and development of different breast cancer subtypes.

### Breast Cancer Drug Targets are Enriched in the Key Dysregulated Network

**FIGURE 5:**
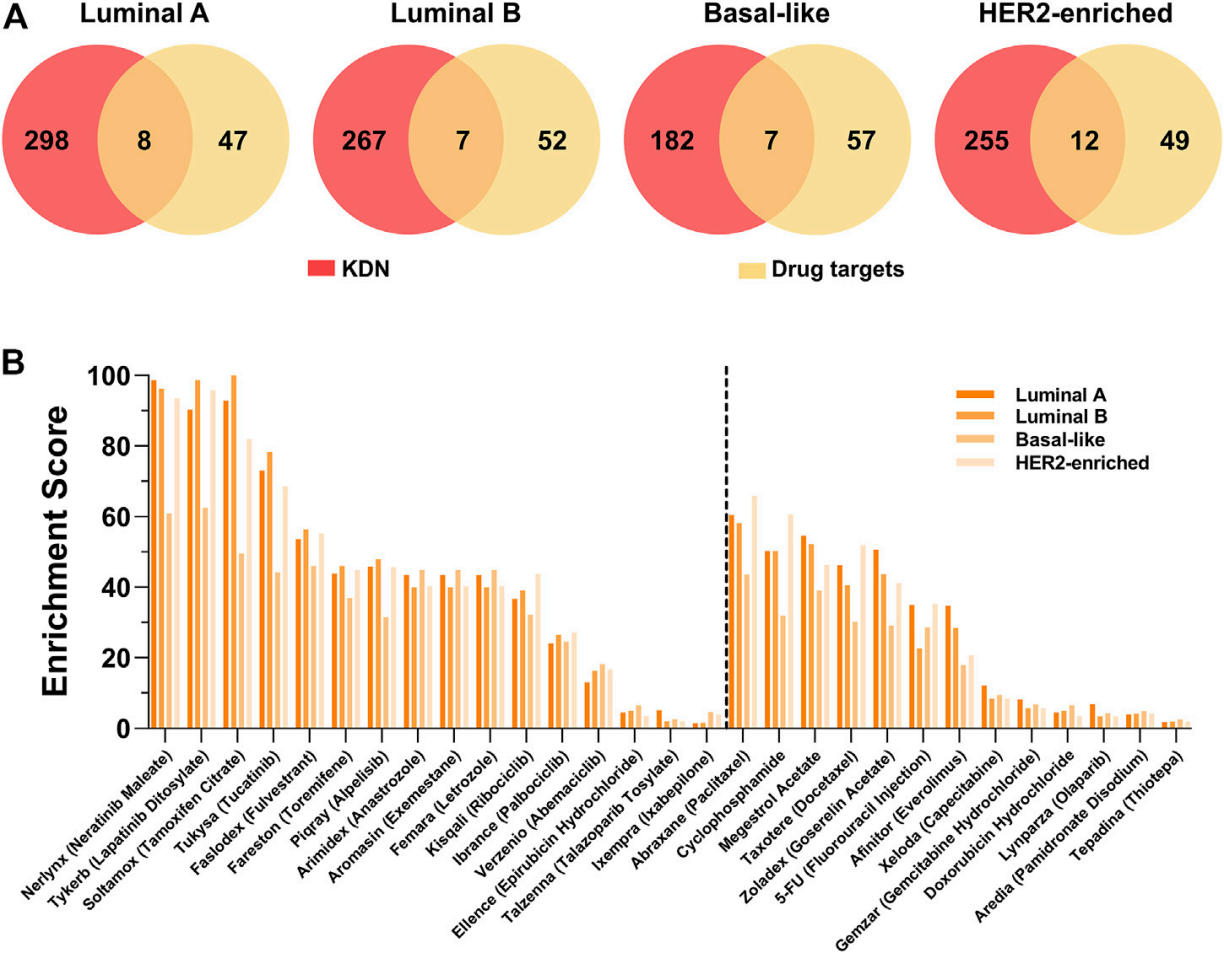
Drug target analysis. **(A)** The Venn graph of the targets of 29 breast cancer drugs and genes in the KDN. **(B)** The enrichment scores of 29 breast cancer targeted drugs.

From the perspective of the targeted therapy, the targets of drugs for breast cancer should be closely related to the KDN. Figure 5A shows the Venn graph of the targets of 29 breast cancer targeted drugs and the key genes. Only a few targets, such as ESR1, ESR2, EGFR, ERBB2, etc. are observed in the KDN. Table 4 lists these targets which are targeted by 10 drugs. As most drugs’ number of targets ranges between 2 and 3, we use their first order neighboring genes to determine the enrichment score. The enrichment score is defined as the negative logarithm of the *p*-value of the hypergeometric test. Figure 5B shows the enrichment scores of the 29 drugs. Drugs solely for breast cancer are on the left, while those that can also treat other cancers are on the right. Obviously, most of the drugs are significantly enriched in the DN. In each DN, these enrichment scores are greater than 2. This demonstrates that the targets of these 29 drugs are closely related to the KDN.

**TABLE 4:** The drug targets in key genes.

Subtypes	Drug targets
Luminal A	ESR1, NR3C1, PRKCG, EGFR, PRKCA, ESR2, PRKCZ, PRKCB
Luminal B	ESR1, NR3C1, PRKCG, EGFR, PRKCA, ESR2, PRKCZ
Basal-like	ESR1, NR3C1, PRKCG, MAPT, PGR, BCL2, ERBB4
HER2-enriched	ESR1, NR3C1, PRKCG, EGFR, PRKCA, ESR2, PRKCZ, ERBB2, MAPT, PGR, BCL2, CYP2A6

To take a further look at the neighbors of targets not observed in the KDN, we find that some neighbors are the targets of other drugs in the KDN. For example, ESR1 and ESR2, targets of Soltamox (Tamoxifen Citrate) and Faslodex (Fulvestrant), are both neighbors of the other six drug targets, such as Nerlynx (Neratinib Maleate), Tykerb (Lapatinib Ditosylate), Abraxane (Paclitaxel), Cyclophosphamide, Megestrol Acetate, and Taxotere (Docetaxel). ESR1 is also a neighbor of CYP19A, which is the target of Arimidex (Anastrozole), Aromasin (Exemestane), and Femara (Letrozole). That is, most drug targets, even if not observed in the KDN, are closely associated with it. Thus, we may hypothesize that the KDN may serve as a critical level point for drugs to exert their effect and to intervene in the abnormal state of the cellular system.

### The Top Dysregulated Genes may Serve as Potential Biomarkers for Survival Analysis

**FIGURE 6:**
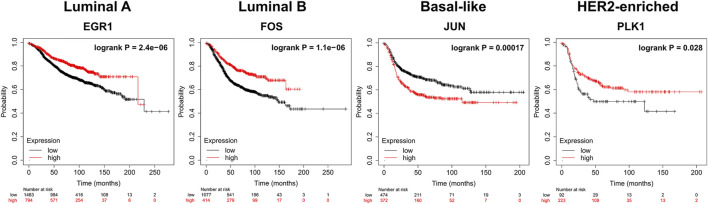
Survival analysis (Kaplan-Meier plots) of dysregulated biomarkers. biomarkers High values are shown in red and low values are shown in black.

We apply KM-plotter to conduct the survival analysis of the top 10 dysregulated genes (http://kmplot.com/analysis/index.php?p=service&cancer=breast). For Luminal A, Luminal B, Basal-like, and HER2-enriched subtypes, 2277, 465, 846, and 315 samples are used respectively. Figure 6 shows the results of the survival analysis with the smallest log-rank *p*-value of gene for each subtype. All *p*-values are less than 0.05. This indicates that these dysregulated genes can be used as potential prognostic markers of breast cancer subtypes.

## Conclusion

From the perspective of biological networks, cancer is a result of the abnormal interactions between genes. In this paper, we propose a simple way to measure the regulation strength of genes based on their relative expression values. And then we construct the key dysregulated network (KDN) for the four subtypes of breast cancer. Our results show that the KDN is significantly enriched in critical breast cancer-related pathways as well as driver genes; closely associated with drug targets; and have significant differences in survival analysis. The key dysregulated genes can also serve as potential driver genes, drug targets, and prognostic markers for subtype identification. In addition, our results indicate that the key dysregulation analysis is more powerful than the traditional DEG analysis. Therefore, the KDN can be applied to other cancer studies, such as the identification of driver genes, drug repurposing, and so on.

## Data Availability

The original contributions presented in the study are included in the article/Supplementary Material, further inquiries can be directed to the corresponding authors.
